# Contrasting off‐line segmentation decisions with on‐line word segmentation during reading

**DOI:** 10.1111/bjop.12482

**Published:** 2021-01-19

**Authors:** Liyuan He, Ziming Song, Min Chang, Chuanli Zang, Guoli Yan, Simon P. Liversedge

**Affiliations:** ^1^ Tianjin Normal University Tianjin China; ^2^ University of Central Lancashire Preston UK

**Keywords:** word segmentation, Chinese reading, eye movements

## Abstract

In two experiments, we investigated the correspondences between off‐line word segmentation and on‐line segmentation processing during Chinese reading. In Experiment 1, participants were asked to read sentences which contained critical four‐character strings, and then, they were required to segment the same sentences into words in a later off‐line word segmentation task. For each item, participants were split into 1‐word segmenters (who segmented four‐character strings as a single word) and 2‐word segmenters (who segmented four‐character strings as 2 two‐character words). Thus, we split participants into two groups (1‐word segmenters and 2‐word segmenters) according to their off‐line segmentation bias. The data analysis showed no reliable group effect on all the measures. In order to avoid the heterogeneity of participants and stimuli in Experiment 1, two groups of participants (1‐word segmenters and 2‐word segmenters) and three types of critical four‐character string (1‐word strings, ambiguous strings, and 2‐word strings) were identified in a norming study in Experiment 2. Participants were required to read sentences containing these critical strings. There was no reliable group effect in Experiment 2, as was the case in Experiment 1. However, in Experiment 2, participants spent less time and made fewer fixations on 1‐word strings compared to ambiguous and 2‐word strings. These results indicate that the off‐line word segmentation preferences do not necessarily reflect on‐line word segmentation processing during Chinese reading and that Chinese readers exhibit flexibility such that word, or multiple constituent, segmentation commitments are made on‐line.

## Background

In English reading, the visual information provided by the spaces between strings of letters to a very significant degree serves the purpose of demarcating word units, thereby optimizing lexical identification (McConkie, Kerr, Reddix, & Zola, 1988; Reichle, Rayner, & Pollatsek, 1999). In this way, words are generally considered to be a basic unit of meaningful information. Words in Chinese reading are also very important and are also generally considered to be a basic meaningful unit of information. Word units have been shown to play an important role in Chinese reading (e.g., Bai, Yan, Liversedge, Zang, & Rayner, 2008; Li, Bicknell, Liu, Wei, & Rayner, 2014; Li, Gu, Liu, & Rayner, 2013; see also Zang, Liversedge, Bai, & Yan, 2011 for a review). Even though the Chinese script is formed from strings of characters with no obvious visual demarcations denoting word boundaries, and words may be comprised of one, two, three, four, or more characters; that is, there is ambiguity as to the characters that comprise a word, native readers have little difficulty processing text. When learning to read Chinese text, children use the alphabetic pinyin system that provides guidance as to the phonological form of Chinese characters in the script. In order that characters may be learned and read effectively, children must establish the relationship between the orthographic and phonological forms for each of the characters they encounter. One might, therefore, assume that the route to establishing lexical representations of words in the lexicon is via the pinyin cues provided during learning, however, this is not the case since pinyin is presented with a space between each pinyin unit (and each unit corresponds to a single character), and the pinyin units are kept uniformly separate, not being grouped into multi‐pinyin ‘word’ units, and therefore providing children no cues to word boundaries. This makes the question of how word boundaries become establishes and how the process of word segmentation develops even more of a puzzle.

It is important to note that, despite these points, word segmentation remains a necessity for effective Chinese reading. And given this, it should be very clear that processes associated with the identification of words and other meaningful units during reading are quite different in Chinese compared to those for alphabetic languages. How Chinese readers break up the continuous string of characters into individual words and potentially, other units to ensure efficient linguistic processing remains a fundamental issue that is not yet fully understood and requires further research.

Quite a number of studies have investigated the text segmentation process in Chinese reading (e.g., Bai et al., 2008; Hoosain, 1992; Liu, Li, Lin, & Li, 2013; see Li, Zang, Liversedge, & Pollatsek, 2015 for a review). Despite the fact that words have been shown to be important in Chinese reading, several studies have also revealed that there can be disagreement between Chinese readers as to the characters that form words. For example, the four‐character string ‘电流强度’ (means *the strength of electric current*) may be considered as one word by some Chinese readers but two words (‘电流’ means *electric current*, and ‘强度’ means *strength*) by others due to lack of a visible boundary between two constituent words. It is also the case that there can often be a lack of clarity with respect to the characteristics that are directly relevant to the concept of a word (e.g., Bassetti, 2005; Hoosain, 1992; Liu, Li, Lin, & Li, 2013), though we note this situation likely pertains to readers of many languages, not just Chinese readers. In off‐line segmentation tasks, the participant is asked to place a vertical line at each word boundary to show where they think each word begins and ends (Hoosain, 1992; Liu et al., 2013). Numerous studies using this methodology have demonstrated that the equivocal nature of the concept of a word makes word segmentation judgments challenging to Chinese readers, and disagreement as to the characters that form a word is common (Bassetti, 2005; Hoosain, 1992; Liu et al., 2013; Peng & Chen, 2004). For example, Liu et al., (2013) required 142 participants to segment Chinese sentences into words and showed that Chinese readers’ segmentations varied according to the syntactic categories of the elements over which they performed the operation. Participants frequently failed to consider auxiliary words, numerals and quantifiers as single word units, whereas adverbs, conjunctions, and prepositions were more often considered to be words in their own right. Moreover, readers tended to combine single character function words with content words to form multi‐word units, being inclined to demark several individual words as a single word unit. Specifically, participants systematically overextended, to combine monosyllables with disyllables to form a ‘word’ supporting the ‘overextension of monosyllable words’ hypothesis, offered earlier by Peng and Chen (2004). Importantly, these conclusions are based on the implicit assumption that off‐line word segmentation preferences reflect the nature of readers’ mental representations of words (or perhaps more accurately, meaningful lexicalized units). Beyond this, data from off‐line word segmentation experiments have also been taken to be indicative of word segmentation strategies that occur during natural reading (e.g., Bai et al., 2008; Li et al., 2015; Zang et al., 2011 for reviews), though very few, if any, studies have investigated whether there are strong correspondences between off‐line word segmentations and lexical representations of Chinese words (or other multi‐constituent units), as well as consequent correspondences between off‐line segmentations and on‐line segmentations that occur during natural reading. Thus, this was one purpose of the present investigation.

Consistent with the suggestion that readers may sometimes process multi‐constituent units as single elements, in a clever experiment, Li, Rayner, and Cave (2009) briefly presented participants with four‐character strings on a computer screen individually in which the four characters formed a single four‐character word (e.g., 不知所措, which means *be at a loss*), 2 two‐character unrelated words (where the words together did not form a meaningful string, e.g., 急速切实, which means *Quickness, feasible*), 2 two‐character related words (where the two words co‐occurred frequently together and were meaningfully related, e.g., 美满婚姻, which means *happy marriage*) and four‐character non‐words (where the four characters were randomly selected and did not make sense together, e.g., 艾抵积促). Participants were asked to report as many characters as possible. Results showed the characters in the second word were reported less often in the related words condition than those in the single word condition but better than those in the 2 two‐character unrelated words condition (and the random character condition). In relation to the present study, these findings suggest that two words can be processed more efficiently when they appear together frequently in text and form a meaningful unit. This, in turn, suggests that with reading experience, smaller word combinations may unite and become represented lexically as a single multi‐constituent unit. Note, though, that the stimuli used in the Li et al. study were selected according to how frequently they co‐occurred in a corpus, and not on the basis of readers’ segmentation judgments. Thus, whilst these data are suggestive of the possibility that readers lexically represent multi‐constituent units, they do not demonstrate this, and nor do they show direct linkage between off‐line segmentation preferences and on‐line processing commitments.

There is some evidence to suggest that English readers may process multi‐constituent units as single elements. For example, Cutter, Drieghe, and Liversedge (2014) examined processing of spaced compounds comprised of two words. In this experiment, the boundary paradigm was adopted with the boundary being positioned prior to the first constituent of the spaced compound. The results from this study showed that parafoveal preview of the second constituent of the compound only occurred when the first constituent was parafoveally available. The authors argued that when the first word was parafoveally available this licensed parafoveal processing of the second constituent and under such circumstances readers processed both words as a single unit. In contrast, when the first word of the spaced compound was not available, preview effects of the second constituent did not occur. To be clear, it appeared that readers operationalized parafoveal processing over the full spaced compound word (i.e., the multi‐constituent unit) when it was parafoveally available in its entirety, which in turn suggests that readers were processing multi‐constituent units as single elements during reading.

Although this study provides some suggestion that readers may process multi‐word units as single elements during reading, it need not necessarily be the case that such processing preferences are reflected in off‐line word segmentation preferences (although such a possibility seems at least plausible). Furthermore, studies such as this provide no attempt to assess the relationship between off‐line segmentation preferences and on‐line segmentation processes during normal reading. To more directly investigate the issue of whether off‐line segmentation preferences reflect on‐line segmentation in reading, in the present study we adopted a somewhat different approach. In Experiment 1, we adopted a relatively simple procedure whereby we asked participants to read sentences that contained a critical four‐character string that could potentially either form a single four‐character word or, instead, could form 2 two‐character words. After participants had read these sentences and we had collected their eye movement data during reading, we then required them to provide off‐line word segmentation judgments on the same sentences that they had just read. Next, to analyse the eye movement data in relation to the off‐line word segmentation data, we split the participants into two groups according to their off‐line segmentation preferences on the critical region (i.e., those that segmented each particular string as a single word, one‐word segmenters; and those that segmented the string as 2 two‐character words, two‐word segmenters). We then analysed the eye movement data for each item based on the participants being split into the two groups of one‐word and two‐word segmenters. Based on the off‐line segmentation data, for every item we knew exactly which participants segmented this as a single word (the one‐word segmenters), and exactly which participants identified this as two words (the two‐word segmenters). Note also that by considering the off‐line segmentation data for each item in this way, the particular participants that formed the one‐word segmenter group and the two‐word segmenter group could (and did) vary from item to item. For Experiment 1 we had a very simple hypothesis, namely, that if readers’ off‐line word segmentation preferences reflected on‐line word segmentation processing during normal reading, then we should observe differences in the eye movement behaviour for the one‐word segmenters compared to the two‐word segmenters on the ambiguous target character string. More specifically, the one‐word segmenters, who we anticipated would process the target string as a single word, would carry out lexical identification only once, whereas the two‐word segmenters should carry out this process twice. On the basis of this logic, we predicted longer reading times, and more fixations on the target string for two‐word segmenters than the one‐word segmenters. Note, though, that we did not predict that eye movement indices for the two‐word segmenters would be double those of the one‐word segmenters because, of course, the one‐word segmenters would carry out lexical identification on a single four‐character word which would take longer (and involve making more fixations) than lexical identification of a two‐character word (Li, Liu, & Rayner, 2011; Zang, Fu, Bai, Yan, & Liversedge, 2018).

## EXPERIMENT 1

Experiment 1 was conducted to explore the extent to which readers’ off‐line segmentation preferences reflected their on‐line segmentation preferences during normal reading.

### Method

#### Partiscipants

Thirty‐four 19 to 25 year old (mean 21.2 years) native Chinese speakers (Tianjin Normal University undergraduates) were paid 25 Yuan (approximately $4) to participate in the experiment. All the participants had normal or corrected‐to‐normal vision.

#### Apparatus

The materials were presented on a 19‐inch CRT monitor (resolution: 1024 × 768 pixels; refresh rate: 120 Hz) connected to a DELL PC. Each sentence was displayed on a single line in Song 21‐point font and the characters were shown in black on a white background. Eye movements were recorded using an SR Research Eyelink 2000 eyetracking system with a sampling rate of 1000 Hz.

#### Materials

**Figure 1:**
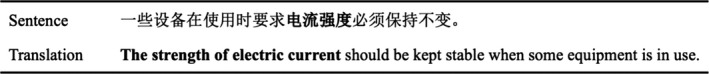
An example of the stimuli used in Experiment 1 (target words are in bold but were not presented in bold in the experiment).

In line with the results of Liu et al. (2013), we identified 150 four‐character strings that included three kinds of 2 two‐character word combinations: adjective noun, noun noun, and adverb adjective combinations. We considered these three types of combinations to be good candidate strings for potential segmentation as a single four‐character word by approximately half of our participants, and as 2 two‐character words by the other half. We then embedded these targets in sentence frames designed not to favour one segmentation over the other (see Figure 1 for an example sentence). We asked fifty undergraduate participants to carry out an off‐line segmentation pre‐screen, inserting vertical lines into each sentence to indicate the boundaries between the words according to their knowledge of Chinese. We then selected 74 sentences from the 150 according to the following criteria: First, for each selected item, the percentage of 1‐word and 2‐word segmentations had to be between 40% and 60%, (*M* = 52.8%, *SD* = 8.4%), that is to say, approximately 50% of participants segmented the target string as one word and approximately 50% as two words. Second, there had to be high agreement regarding the word boundary before the target string, 92.1% (*SD* = 5.3%), and at the end of the target string, 89.6% (*SD* = 4.5%). That is to say, almost all the participants agreed on where the target string began and ended. Thus, word boundary ambiguity was almost entirely due to whether the target string was a single four‐character word, or 2 two‐character words. This selection procedure allowed us to be confident that for each item we would have two participant groups, each group having an approximately similar number of participants, with each group segmenting the target character string differently.

All the sentences were rated on a 5‐point scale by 24 university students for their naturalness. These participants did not take part in the eye‐tracking study. The mean naturalness score was 4.2 (*SD* = 0.39) (where a score of 5 was ‘very natural’). The contextual predictability of the target words was assessed by 21 undergraduates who also did not take part in the eye‐tracking experiment. They were given the sentence fragment up to but not including the target strings and were then asked to complete the sentence fragment. The mean predictability for the target strings was calculated as the percentage of participants who provided the target string within all the participants, and the value was very low (*M* = 8%). The length of all the sentences ranged from 18–22 characters.

#### Procedure

There were two tasks in this experiment: An on‐line reading task and an off‐line segmentation task. Participants first carried out the reading task and then they carried out the segmentation task.

##### Reading task

Participants were tested individually. After entering the laboratory, participants were seated 63 cm from a video monitor. At this viewing distance, each character subtended a visual angle of 0.8°. A chin/forehead rest was used to minimize head movements. Viewing was binocular, but eye movement data were only collected from the right eye. At the beginning of the first task, we presented participants with instructions and then asked them to perform a calibration procedure by looking at a sequence of three fixation points displayed in a random order horizontally across the middle of the computer screen. Following calibration, the gaze position error was smaller than 0.2° of visual angle. At the beginning of each trial, a black circle (about 0.8° × 0.8°) appeared on the left side of the computer screen, indicating the position of the first character in the sentence. Once the participant had fixated the black circle successfully, a sentence was presented. Participants were instructed to read and comprehend the sentences silently and then to occasionally answer questions about the sentences. The comprehension questions were presented after approximately one third of the sentences. After reading a sentence, the participants were asked to press a response button to start the next trial. This part of the experiment took approximately 30 min.

##### Word segmentation task

After the participant had finished reading the sentences, we first asked them to write down their concept of a Chinese word based on their knowledge. After this, we gave them the sentences from the reading task printed on sheets of paper and asked them to segment the normal Chinese sentences by placing a slash between each of the words. This task took approximately 30 min.

### Results and discussion

First, we considered all of the responses that participants provided in respect of their concept of a word and we found that these expressed ideas quite similar to those features reported by Liu et al. (2013). Summarily, participants considered words to be small, independent, meaningful units of text. At a general level, it is clear from the findings of Liu et al., and from the responses in our study that a Chinese reader’s concept of a word is vague.

Next, we focused our analyses on the critical four‐character target string. First, we analysed the data from the word segmentation task in which we considered each item separately, and for each item, we split the participant group into two, based on their off‐line word segmentation preferences. Two kinds of segmentation were taken into consideration when undertaking our analyses. First, it was necessary that a participant placed a slash at the beginning of the target character string and at the end of the target character string. If the participant did not place segmentation lines at these two positions or between the 1st and 2nd or the 3rd and 4th characters, this item for that participant was excluded from further data analysis (5.6% of the trials). Second, we assessed whether a participant did, or did not, place a line between the second and the third character of the target character string. If a participant did make such a segmentation they were categorized as a two‐word segmenter for that item, and if they did not, they were categorized as a one‐word segmenter. We then analysed the eye movement data for each item based on the categorization of each of the participants for that item as a one‐word, or two‐word segmenter.

Accuracy on the comprehension questions was high (92.3%), indicating that participants understood the sentences well. Trials in which participants blinked more than three times, or blinked once when they fixated the target character string, were excluded from the analyses, resulting in the loss of a further 3.8% of the trials. Fixations with durations longer than 1200 ms or shorter than 80 ms (3.2% of all fixations) were also excluded from the analyses.

Analyses were carried out on three regions: (1) the four character target region, (2) the first two characters of the target region (word 1), and (3) the last two characters of the four character target region (word 2). For these three regions, we computed first fixation duration (FFD, the duration of the first fixation on the target region, irrespective of the number of fixations made on that region); gaze duration (GD, the sum of all fixation durations on the target region before the eyes moved to another region); single fixation duration (SFD, the duration of a fixation when readers made only one fixation on the region before the eyes moved to another region), the skipping probability (SP, the likelihood that readers skip a word during first pass reading), the number of first pass fixations (NFPF, the number of fixations made during first pass reading on the target word), and total reading time (TT, the sum of all fixations on a word, including fixations made after regressions) (see Rayner, 1998, 2009).

To analyse these data, we computed linear mixed‐effects models using the lme4 package (Bates, Maechler, & Bolker, 2015) in R 3.5.2 (R Development Core Team, 2018) and R studio 1.1.463 (2018). This type of analysis is ideal for analysing the data from the current experiment in which different participants’ segmentation preferences might vary for different critical strings. Group (one‐word segmenters and two‐word segmenters) was treated as a fixed factor, participants and items were treated as crossed random factors. The fixation time analyses were carried out on log‐transformed data to increase normality. The skipping data were analysed using logistic models due to the binary nature of the variable. To maximize the generalizability of our analyses, we used the maximal random‐effects structure (Barr, Levy, Scheepers, & Tily, 2013) for all measures. If the maximum random model did not converge, the model was trimmed starting with removal of correlations between factors, then random factors for items until the model converged (the models for each measure were computed using R scripts, and these are provided on‐line: see Open Science Framework, https://osf.io/dsf32/). Table 1 shows the means and standard deviations for the whole four‐character target region, word 1 and word 2. The beta values from the models are shown in Table 2. As we can see, for all measures, there were no significant differences between the one‐word segmenter and the two‐word segmenter groups when they processed the four character region, word 1 or word 2^1^.

**Table 1:** Eye movement measures for target regions in experiment 1

	FFD	GD	SFD	NFPF	TT	SP
The whole four‐character region						
1‐Word Segmenter	250 (82)	488 (256)	245 (90)	1.96 (.90)	788 (469)	.02 (0.15)
2‐Word Segmenter	239 (78)	448 (237)	238 (86)	1.88 (.88)	733 (436)	.03 (0.16)
First 2‐Character region						
1‐Word Segmenter	248 (80)	287 (132)	249 (80)	1.15 (0.37)	443 (273)	.17 (.38)
2‐Word Segmenter	239 (78)	269 (115)	239 (76)	1.13 (0.34)	415 (246)	.19 (.40)
Last 2‐Character region						
1‐Word Segmenter	248 (83)	283 (124)	247 (80)	1.15 (0.38)	417 (257)	.19 (.39)
2‐Word Segmenter	241 (80)	275 (121)	239 (79)	1.14 (0.35)	389 (234)	.21 (.41)

Note

FFD = first fixation duration; GD = Gaze duration; NFPF = number of fixations in the first pass; SFD = single fixation duration; TT = total fixation duration; SP = skip probability.

**Table 2:** Fixed and random effects from the linear mixed model analyses on target regions in Experiment 1

Fixed effects	FFD			GD			SFD			NFPF			TT			SP		
	B	SE	t	b	SE	t	b	SE	t	b	SE	t	b	SE	t	b	SE	z
*The whole four‐character region*																		
Intercept	5.45	0.02	**287.39**	6.00	.04	**158.22**	5.41	.03	**180.24**	0.54	.03	**16.98**	6.46	.05	**122.88**	**‐4.38**	.32	**‐13.56**
Group Effect	−0.01	0.02	−0.67	0.01	.03	0.24	−0.02	.03	0.49	0.00	.03	0.06	0.01	.03	0.36	−0.03	.47	−0.07
*First 2‐character region*																		
Intercept	5.45	0.02	**297.82**	5.55	.02	**251.46**	5.45	.02	**290.05**	0.09	.01	**8.41**	5.88	.04	**156.38**	−1.63	.12	**−13.35**
Group Effect	−0.02	0.02	−1.25	−0.03	.02	−1.31	−0.02	.02	−1.02	0.00	.01	0.08	−0.03	.03	−0.99	0.10	.13	0.77
*Last 2‐character region*																		
Intercept	5.45	0.02	**270.83**	5.54	.02	**234.83**	5.44	.02	**278.09**	0.09	.01	**7.13**	5.82	.04	**136.48**	−1.59	.15	**−10.50**
Group Effect	0.01	0.02	0.51	0.02	.02	0.90	−0.00	.02	−0.04	0.01	.01	0.48	−0.01	.03	−0.31	−0.06	.15	−0.38

Random effects	Var	SD	Var	SD	Var	SD	Var	SD	Var	SD	Var	SD
*The whole four‐character region*												
Item	0.00	0.05	0.02	0.13	0.00	0.06	0.01	0.09	0.04	0.20	0.20	.44
Participant	0.01	0.10	0.04	0.19	0.02	0.15	0.03	0.17	0.07	0.27	1.27	1.13
Residual	0.10	0.31	0.26	0.51	0.11	0.33	0.17	0.41	0.25	0.50	–	–
*First 2‐character region*												
Item	0.00	0.05	0.00	0.06	0.00	0.05	0.00	0.02	0.02	0.13	0.16	.39
Participant	0.01	0.09	0.01	0.11	0.01	0.09	0.00	0.05	0.04	0.19	0.31	.56
Residual	0.10	0.31	0.14	0.38	0.09	0.30	0.05	0.23	0.26	0.51	−	−
*Last 2‐character region*												
Item	0.00	0.06	0.01	0.10	0.00	0.06	0.00	0.04	0.03	0.17	0.14	.37
Participant	0.01	0.10	0.01	0.11	0.01	0.10	0.00	0.06	0.04	0.21	0.59	.77
Residual	0.10	0.32	0.15	0.38	0.96	0.31	0.06	0.23	0.24	0.49	−	−

Note

Significant items are presented in bold.

FFD = first fixation duration; GD = gaze duration; NFPF = number of fixations in the first pass; SFD = single fixation duration; SP = skipping probability; TT = total fixation duration.

As is probably obvious, the lack of effects in Experiment 1 was completely unexpected to us. Recall, we predicted that by dividing our participant groups on the basis of their off‐line word segmentation preferences for each individual item, we would be able to observe systematic, corresponding differences in their on‐line reading behaviour. We expected that the one‐word segmenters for each item would process that target string in a sentence as a single unit, and therefore make fewer fixations and have shorter reading times than the two‐word segmenters who would process the same string as two separate units. However, there was no suggestion of any such differences, and to reiterate, we found this quite surprising. In order to provide statistical support for the null effects, we undertook Bayes factor analyses for our linear mixed models (Morey, Rouder, Jamil, Urbanek, Forner, & Ly, 2018) in relation to all the measures. Bayes factors for the full model (i.e., *BF_Full_*, the model containing the main effects of group) and the model without group effects (i.e., *BF_Base_*) were calculated. We were able to evaluate the null effect of group by comparing the Full model and Base model (BF = *BF_Full_*/*BF_Base_*). BF values smaller than 1 favour the null hypothesis, whereas BF values greater than 1 favour the alternative hypothesis (Abbott & Staub, 2015). For each of the measures, we used the default scale prior (*r* = .5) and 100,000 Monte Carlo iterations of the BayesFactor package. The results of the Bayesian analyses supported the null hypothesis. A sensitivity analysis with different priors (i.e., 0.25, 0.5 and 0.1) provided consistent results. The results of the Bayesian analyses are shown in Table 3.

**Table 3:** Bayesian factor values for each of the measures for the target region in experiment 1

Measures	FFD			GD			SFD			NFPF			TT			SP		
Priors	.25	.5	1	.25	.5	1	.25	.5	1	.25	.5	1	.25	.5	1	.25	.5	1
The whole four‐character region																		
Group effect	.16	.09	.04	.13	.07	.03	.20	.11	.06	.13	.07	.03	.13	.07	.03	.11	.06	.01
First 2‐Character region																		
Group effect	.34	.17	.06	.27	.13	.06	.25	.12	.01	.12	.06	.02	.18	.10	.05	.15	.08	.06
Last 2‐Character region																		
Group effect	.15	.08	.04	.06	.10	.07	.14	.07	.05	.15	.08	.03	.13	.07	.03	.17	.06	.01

Note

FFD = first fixation duration; GD = Gaze duration; SFD = single fixation duration; NFPF = number of fixations in the first pass; TT = total fixation duration; SFD = single fixation duration; SP = skipping probability.

Given the very robust contrasting effects that we observed in the off‐line segmentation data relative to the on‐line eye movement data, in our view, these null effects are extremely interesting and are consistent with the suggestion that differences in off‐line segmentation preferences do not necessarily reflect on‐line segmentation preferences during reading. An alternative possibility, however, is that in Experiment 1 we did not identify our participant groups, nor our stimuli such that both together afforded the possibility of observing the effects that we predicted. We consider this issue in more detail below.

## EXPERIMENT 2

The results of Experiment 1 left us with almost more questions than answers with respect to the relationship between off‐line segmentation preferences and on‐line segmentation during reading. One obvious conclusion that we may form is that patterns of effects in off‐line segmentation data do not necessarily reflect on‐line segmentation differences that occur during normal reading. However, before we accept this suggestion we need to consider carefully whether the selection criteria for our stimuli in Experiment 1 were adequate to provide a strong test of our hypothesis, and whether the way we split our participant groups with respect to their segmentation preferences for particular items gave us the best chance of obtaining robust effects. In the off‐line segmentation data, the participants in Experiment 1 exhibited a 1‐word segmentation preference approximately 50% of the time, and a 2‐word segmentation preference 50% of the time.

**Figure 2:**
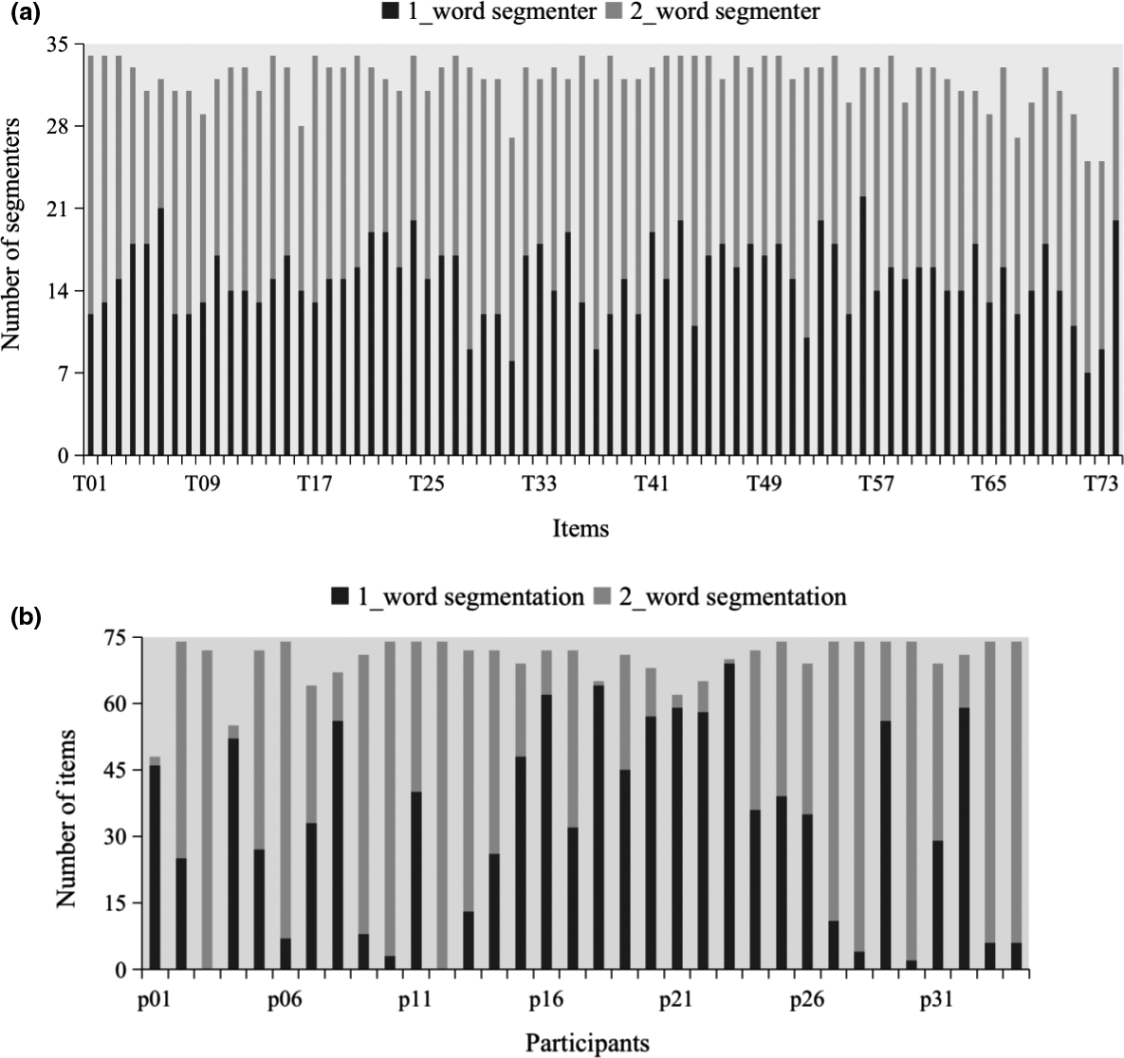
The distributions of segmentations for items and participants for the four‐character strings in the segmentation test conducted after the eye‐tracking testing session in Experiment 1. Panel A shows the distribution of segmentations for the four‐character strings in relation to the item population for the segmentation test. The horizontal axis shows the items (74 in total), and the vertical axis shows the number of segmenters who segmented each four‐character string in each item into either a single four‐character word, or two two‐character words (34 participants in total). Panel B shows the distribution of segmentations for the four‐character strings in relation to the participant population for the segmentation test. The horizontal axis shows the participants (34 in total), and the vertical axis shows the number of items that were segmented as either a single four‐character word, or two two‐character words (74 items in total). Note that some participants did not place segmentation lines between the 1st and 2nd or the 3rd and 4th characters for some items, and these items for that participant were excluded from the analysis.

For each stimulus that we used in Experiment 1, the off‐line segmentation data demonstrated that each was preferentially segmented as a single four‐character word on half of the trials, and as 2 two‐character words on the remainder. The response distribution of segmentations for the four‐character strings in relation to the item and participant populations tested in the segmentation test carried out after the eye‐tracking testing session in Experiment 1 are shown in Figure 2. (These data are also provided on‐line: see Open Science Framework, https://osf.io/dsf32/). Thus, in Experiment 1 we tested a group of stimuli and participants that were fundamentally heterogeneous with respect to segmentation (items were segmented each way about half of the time, and participants segmented items each way about half of the time).

To avoid such heterogeneity in Experiment 2, it was necessary to select both our participant groups and our stimuli in a slightly different way. We carried out a larger scale pre‐screen study in which we selected our 1‐word segmenters and our 2‐word segmenters such that each group had a very consistent bias to segment strings as a single word, or 2 two‐character words respectively for almost all the stimuli that we used in the pre‐screen. Furthermore, we used a greater number of stimuli in the pre‐screen study to allow us to also select a set of target strings that almost all participants consistently identified as being a single four‐character word, and a set that were consistently identified as being 2 two‐character words. In this way, unlike in Experiment 1, in Experiment 2 we used two stimulus sets for which there was a consistent segmentation bias across the majority of items (i.e., a homogeneous set of stimuli for which there was a consistent one‐word segmentation bias, and a homogeneous set of stimuli for which there was a consistent two‐word segmentation bias). In addition to the selection of these two sets of stimuli, we selected a third set of stimuli that were purposefully ambiguous. That is, we selected a third set of stimuli that were segmented as a single word by half the participants and as 2 two‐character words by the remaining participants. Finally, in Experiment 2, we also identified two participant groups, for one of which all participants exhibited a consistent bias to segment the target as a single word, whilst for the other there was a consistent bias to segment the target as 2 two‐character words (i.e., two homogeneous participant groups each with a different segmentation bias).

By comparing eye movement behaviour during reading across the two participant groups for each of these stimulus sets, we were able to explore how readers segmented text containing target character strings that were unambiguously formed of a single four‐character word or 2 two‐character words, and assess how they processed strings that were ambiguous between these two forms.

### Method

#### Participants

Before the experiments, we carried out a pre‐screen test in which we tested a new group of 100 participants in a ‘constituent decision’ task. In this task, participants were presented with a four‐character string and were required to make a judgment as to whether the four characters were a single four‐character word, or 2 two‐character words. We presented participants with 190, four‐character strings. To select the stimuli for this pre‐screen, we used the results from Liu et al. (2013) for guidance. Thirty percent of the four‐character strings were judged likely to be categorized as a single four‐character word, 30% were judged likely to form 2 two‐character words, and the final set of strings (40%) were judged likely to be ambiguous between the two. We identified two groups of participants based on the percentage of ‘single four‐character word’ vs. ‘2 two‐character word’ responses each participant made. We selected a group of 13 participants who almost always judged the four‐character strings to be a single word (*M* = 83.1%, *SD* = 4.6%), and a second group of 12 participants who very frequently judged the four‐character strings as 2 two‐character words (*M* = 72.1%, *SD* = 6.9%). All the participants were undergraduates from Tianjin Normal University (mean age 21.4yrs), had normal or corrected‐to‐normal vision, and were naive to the purpose of the experiment. None of these participants took part in Experiment 1. They were paid 10 Yuan (approximately $1.5) to participate.

#### Apparatus

The apparatus was identical to Experiment 1.

#### Materials

**Figure 3:**
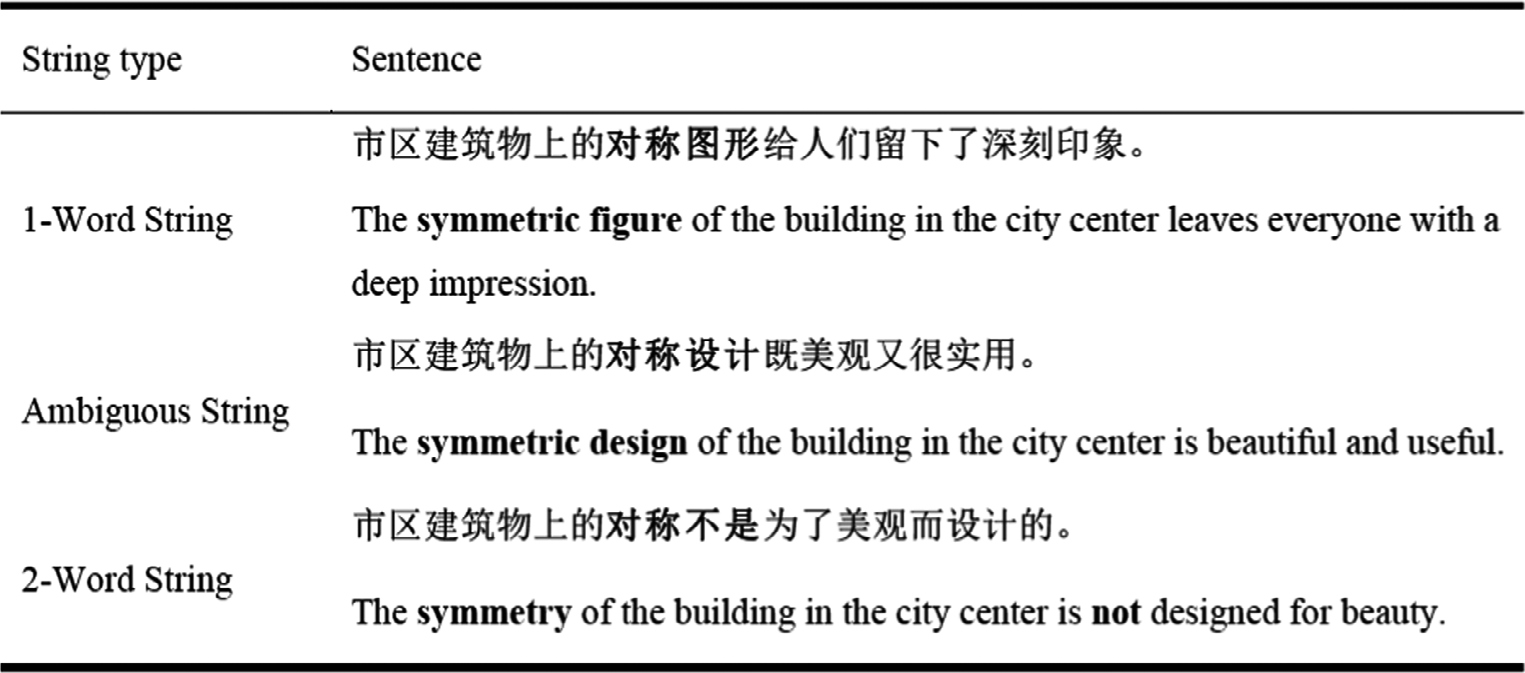
An example of the stimuli used in Experiment 2 (target words are in bold but were presented normally in the experiment).

In order to select the stimuli for the current experiment, we carried out a second constituent decision task pre‐screen test. Fifty participants who did not take part in the earlier constituent decision task were tested. We first constructed 113 sets of character string triplets, with each triplet being comprised of four characters. The four characters could be categorized as (i) predominantly a single, four‐character word; (ii) predominantly 2 two‐character words; (iii) approximately equally often classed as a single four‐character word and as 2 two‐character words. The four‐character strings that comprised each triplet shared the same first two characters (i.e., the first word), and the final two characters that differed across triplets were matched on lexical frequency as well as stroke complexity (see Table 4). To reiterate, for each triplet there was one form that we expected would be segmented as a single word, another that we expected would be segmented as 2 two‐character words, and a final form that we expected to be ambiguous such that approximately half of the pre‐screen participants would categorize it as a single four‐character word, whilst the others would categorize it as 2 two‐character words (see Figure 3).

**Table 4:** Properties of the critical words in experiment 2

	Frequency (per million)	Complexity (Stroke number)	Predictability (%)	Critical string predictability (%)	Sentence naturalness
1‐Word String	94 (131)	15.21 (3.72)	21.1 (3.1)	0 (0)	6.3 (.3)
Ambiguous String	88 (131)	15.47 (3.61)	0 (0)	0 (0)	5.7 (.4)
2‐Word String	89 (132)	15.70 (3.28)	0 (0)	0 (0)	5.8 (.3)

Note

The Frequency and Stroke Complexity of the final two characters of the three forms of the four character strings; Predictability (%) = the likelihood of the second 2‐character word being predicted based on the first 2‐character word; Critical String Predictability (%) = the likelihood of the target string being produced given the preceding sentence context; Naturalness scores were out of 7, where 7 was very natural.

In a pencil and paper task, fifty participants were required to indicate the word boundaries within each four‐character string presented in a randomized list. On the basis of participants’ segmentations, 57 triplet stimuli were identified for which participants most often segmented the first instance of the triplet as a single four‐character word (*M* = 85.38%), the second instance as 2 two‐character words (*M* = 88.20%), and for the final instance, approximately half the participants segmented it as a four‐character word, with the remaining participants segmenting it as 2 two‐character words (four‐character segmentation *M* = 57.64%).

We embedded these stimuli into 57 sentence frames in which the characters prior to the critical string were the same. Sentences ranged from 18–24 characters in length. All the sentences were rated on a 7‐ point scale for their naturalness (where a score of 7 was ‘very natural’) by 20 university students who did not take part in any other pre‐screens, segmentation tasks or experiments. There was no significant difference in naturalness across conditions (*F* < 1). As in Experiment 1, we also conducted two sets of predictability assessments for the critical string, each using 15 different participants, none of whom took part in other experiments. In the first, participants were given the sentence fragment up to but not including the critical string and were asked to complete the sentence fragment. No participant produced any of the critical strings, indicating the predictability of all the critical strings was low (see Table 4). In a second predictability assessment, we truncated the sentences after the first two shared characters of the critical string to determine how likely participants were to complete the critical string in one of the three forms we had selected given the first two characters. Unsurprisingly, we found that participants were more likely to complete the critical string as a single four‐character word than either of the two alternative forms. This result indicates that strings selected as being likely four‐character words were more readily available as completions than completions that would represent distinct and independent pairs of two character words. The predictability of the second 2‐character word based on the first was included as a covariate to rule out its possible influences on our dependent measures^2^. All the mean predictabilities and naturalness scores are presented in Table 4.

#### Procedure

The procedure was identical to the reading task in Experiment 1.

### Results and discussion

#### Accuracy

The mean overall comprehension accuracy for the experiment was 91% (*SD *= 0.05), indicating that readers read and understood the sentences.

#### Eye movement results

We undertook the same methods of data treatment in Experiment 2 as were used in Experiment 1. Trials in which participants made three or more blinks were excluded from the analyses, resulting in a loss of 1.8% of the trials. Fixations with durations longer than 1200 ms or shorter than 80 ms (approximately 3.6% of all fixations) were also excluded from the analyses. We report the same eye movement measures as in Experiment 1. Means and standard deviations for the eye movement measures for the target region are shown in Table 5.

**Table 5:** Eye movement measures for target regions in experiment 2

		FFD	GD	SFD	NFPF	TT	SP
The whole four character region							
1‐Word segmenters	1‐Word Strings	250 (87)	444 (228)	254 (101)	1.77 (.77)	637 (376)	.04 (.19)
	Ambiguous Strings	245 (80)	528 (326)	238 (75)	2.10 (1.11)	768 (447)	.02 (.14)
	2‐Word Strings	255 (84)	542 (325)	270 (135)	2.05 (1.16)	862 (548)	.03 (.18)
2‐Word segmenters	1‐Word Strings	242 (72)	446 (241)	255 (107)	1.83 (.87)	723 (432)	.03 (.17)
	Ambiguous Strings	244 (77)	513 (305)	250 (93)	2.06 (1.10)	853 (497)	.02 (.13)
	2‐Word Strings	254 (90)	511 (325)	251 (81)	1.98 (1.10)	860 (481)	.02 (.13)
First 2‐Character region							
1‐Word segmenters	1‐Word Strings	251 (90)	280 (142)	249 (91)	1.10 (0.35)	381 (232)	.22 (.42)
	Ambiguous Strings	247 (81)	308 (166)	249 (85)	1.25 (0.54)	427 (257)	.19 (.39)
	2‐Word Strings	257 (94)	295 (137)	254 (89)	1.18 (0.40)	458 (279)	.23 (.42)
2‐Word Segmenters	1‐Word Strings	247 (82)	285 (133)	247 (83)	1.13 (0.34)	416 (248)	.25 (.44)
	Ambiguous Strings	241 (82)	281 (120)	243 (85)	1.16 (0.42)	440 (286)	.21 (.41)
	2‐Word Strings	250 (91)	293 (145)	247 (88)	1.17 (0.39)	448 (271)	.22 (.41)
Last 2‐Character region							
1‐Word segmenters	1‐Word Strings	251 (87)	289 (130)	253 (88)	1.14 (0.38)	369 (241)	.24 (.43)
	Ambiguous Strings	264 (96)	307 (145)	263 (99)	1.17 (0.46)	420 (264)	.18 (.38)
	2‐Word Strings	268 (109)	335 (181)	262 (102)	1.28 (0.54)	499 (331)	.22 (.42)
2‐Word segmenters	1‐Word Strings	246 (71)	288 (137)	245 (69)	1.14 (0.44)	428 (268)	.23 (.42)
	Ambiguous Strings	276 (111)	324 (161)	279 (117)	1.17 (0.39)	516 (292)	.20 (.40)
	2‐Word Strings	272 (101)	313 (138)	272 (101)	1.21 (0.46)	522 (284)	.21 (.41)

Note

FFD = first fixation duration; GD = Gaze duration; SFD = single fixation duration; NFPF = number of fixations in the first pass; TT = total fixation duration; SP = skipping probability.

We constructed linear mixed‐effects models using the lme4 package (Bates et al., 2015) in R to analyse the data. Group (one‐word segmenters and two‐word segmenters) and Type (one‐word strings, ambiguous strings, two‐word strings) (note, Group and Character Position in the additional analysis) were treated as fixed factors, and an interaction term was included. Participants and items were treated as crossed random factors. Once again, to maximize the generalizability of our analyses, the maximal random‐effects structure was run for all measures. If the maximum random model did not converge, the model was trimmed starting with removing correlations between factors, then interactions, then random factors, also the trimming was first done for items then for participants until the model converged. The successive contrasts were carried out as we compared the one‐word condition with the ambiguous string, the one‐word condition with the two‐word condition, and the ambiguous string with the two‐word condition. Fixed effect estimations for the eye movement measures are shown in Table 6.

**Table 6:** Fixed and random effects from the linear mixed model analyses for each of the measures on the target regions in Experiment 2.

Fixed effects		FFD			GD			SFD			NFPF			TT			SP		
		*b*	*SE*	*t*	*b*	*SE*	*t*	*b*	*SE*	*t*	*b*	*SE*	*t*	*b*	*SE*	*t*	*b*	*SE*	*z*
*The whole four‐character region*																			
Intercept		5.47	.05	**117.41**	6.03	.06	**102.57**	5.47	.02	**238.78**	0.54	.05	**10.53**	**6.48**	.06	**101.81**	−4.07	.36	**−11.15**
Group	Two‐ vs. one‐word segmenters	−0.00	.00	−0.80	−0.02	.11	−0.18	−0.01	.04	−0.21	−0.01	.10	−0.07	.09	.12	0.77	−0.24	.55	−0.44
Covariable	Predictability	−0.01	.01	−1.00	0.09	.09	0.99	−0.09	.09	−1.00	0.03	.08	0.41	0.06	.10	0.62	−0.04	.90	−0.05
String type	One‐word vs. ambiguous	−0.00	.00	**−2.03**	0.15	.04	**3.72**	−0.05	.04	−1.10	0.13	.04	**2.94**	0.21	.04	**5.09**	−0.62	.47	−1.31
	Ambiguous vs. two‐word	0.00	.00	1.51	−0.01	.03	−0.19	0.05	.04	1.38	−0.05	.05	−1.03	0.05	.04	1.45	.25	.46	0.55
	One‐word vs. two‐word	0.00	.00	−0.77	0.14	.04	**3.56**	0.01	.04	0.14	0.08	.04	**1.88^§^**	0.26	.04	**6.28**	−0.36	.45	−−0.55
Interaction	Group × one‐word vs. ambiguous	0.00	.00	−0.39	−0.03	.07	−0.38	0.00	.08	0.05	−0.05	.08	−0.63	0.00	.07	0.01	0.03	.86	0.03
	Group × ambiguous vs. Two‐word	−0.00	.00	−0.35	−0.04	.07	−0.56	−0.05	.08	−0.71	−0.01	.09	−0.06	0.05	.07	−0.77	−0.51	.93	−0.55
	Group × one‐word vs. two‐word	0.00	.00	−0.73	−0.06	.07	−0.93	−0.05	.07	−0.69	−0.05	.07	−0.76	−0.05	.07	−0.76	−0.48	.82	−0.59
*First 2*‐*character region*																			
Intercept		5.46	.02	**245.76**	5.74	.03	**184.88**	5.47	.02	**242.80**	0.10	.02	**5.31**	5.88	.05	**129.03**	−1.57	.21	**−7.56**
Group	Two‐vs. one‐word segmenters	−0.03	.04	−0.70	−0.03	.06	−0.50	−0.03	.04	−0.70	−0.01	.04	−0.41	0.02	.08	0.30	0.13	.38	0.34
Covariable	Predictability	−0.11	.07	−1.63	−0.14	.08	**−1.78^§^**	−0.13	.07	**−1.88^§^**	−0.04	.05	−0.78	−0.10	.10	−0.93	0.69	.43	1.59
String type	One‐word vs. ambiguous	−0.03	.03	−1.2	0.01	.03	0.32	−0.02	.03	−0.80	0.05	.02	**2.10**	0.07	.04	1.55	−0.10	.19	−0.54
	Ambiguous vs. two‐word	0.02	.02	0.96	−0.00	.03	−0.13	.01	.03	0.32	−0.02	.02	−0.71	0.04	.04	1.14	0.19	.17	1.15
	One‐word vs. two‐word	0.01	.03	−0.32	0.01	.03	0.21	−0.02	.03	0.52	0.03	.02	1.16	.11	.04	**2.50**	−0.09	.19	−0.46
Interaction	Group × one‐word vs. ambiguous	−0.00	.05	−0.09	−0.08	.06	−1.35	−0.02	.05	−0.39	−0.07	.04	**−1.77^§^**	−0.04	.07	−0.58	−0.10	.33	−0.28
	Group × ambiguous vs. Two‐word	−0.02	.05	−0.32	0.04	.06	−0.62	−0.02	.05	−0.37	0.04	.05	0.89	−0.03	.07	−0.43	−0.27	.34	−0.79
	Group × one‐word vs. two‐word	−0.02	.05	−0.40	−0.04	.06	−0.72	−0.02	.05	−0.37	−0.03	.04	0.99	−0.08	.08	−0.1.00	−0.37	.33	−1.10
*Last 2*‐*character region*																			
Intercept		5.50	.02	**264.61**	5.62	.03	**15.14**	5.50	.02	**265.75**	0.11	.02	**4.98**	5.93	.05	**126.59**	−1.47	1.89	**−7.83**
Group	Two− vs. one‐word segmenters	0.02	.04	0.61	0.01	.06	0.23	0.03	.04	0.68	−0.01	.04	−0.21	0.14	.09	1.60	0.00	.36	0.00
Covariable	Predictability	0.05	.07	0.76	0.07	.08	0.89	0.02	.07	0.32	0.05	.05	1.10	0.18	.10	**1.85^§^**	−0.41	.42	−0.97
String type	One‐word vs. ambiguous	0.07	.03	**2.35**	0.09	.04	**2.44**	0.06	.03	**1.74^§^**	0.03	.03	1.19	0.19	.04	**4.52**	−0.43	.19	**−2.24**
	Ambiguous vs. two‐word	−0.00	.03	−0.06	0.02	.03	0.70	−0.00	.03	−0.13	0.05	.02	**1.83^§^**	−0.09	.04	**2.40**	0.22	.17	1.32
	One‐word vs. two‐word	0.07	.03	**2.29**	0.11	.04	**3.02**	0.05	.03	**1.67^§^**	0.08	.02	**3.24**	0.28	.04	**6.56**	−0.20	.19	−1.09
Interaction	Group × one‐word vs. ambiguous	−0.04	.05	0.70	0.05	.06	0.77	0.05	.06	0.90	0.02	.05	0.44	0.07	.07	0.92	0.26	.34	−0.77
	Group × Ambiguous vs. Two‐word	−0.01	.05	−0.22	−0.08	.06	−1.26	−0.01	.06	−0.16	−0.06	.05	−1.18	−0.13	.07	−1.74	−0.23	.34	−0.66
	Group × one‐word vs. Two‐word	0.02	.05	0.47	−0.03	.06	−0.47	0.04	.06	0.79	−0.03	.04	−0.79	−0.06	.07	−0.81	0.03	.33	0.11

Random effects	*Var*	*SD*	*Var*	*SD*	*Var*	*SD*	*Var*	*SD*	*Var*	*SD*	*Var*	*SD*
*The whole four*‐*character region*												
Item	0.12	0.35	0.01	0.12	0.00	0.00	0.01	0.10	0.03	0.17	0.00	0.03
Participant	0.00	0.00	0.07	0.27	0.01	0.07	0.06	0.24	0.08	0.29	0.88	0.94
Residual	0.00	0.03	0.26	0.51	0.12	0.35	0.16	0.40	0.27	0.52	−	−
*First 2*‐*character region*												
Item	0.00	0.07	0.00	0.07	0.00	0.06	0.00	0.03	0.02	0.14	0.27	0.52
Participant	0.01	0.09	0.02	0.13	0.01	0.09	0.01	0.08	0.04	0.19	0.76	0.87
Residual	0.11	0.33	0.16	0.41	0.11	0.32	0.06	0.25	0.28	0.53	−	−
*Last 2*‐*character region*												
Item	0.00	0.05	0.00	0.04	0.00	0.06	0.00	0.02	0.01	0.11	0.11	0.33
Participant	0.01	0.08	0.02	0.13	0.01	0.07	0.01	0.10	0.04	0.21	0.66	0.81
Residual	0.12	0.35	0.17	0.42	0.11	0.34	0.07	0.26	0.27	0.52	−	−

Note

Significant items are presented in bold.

FFD = first fixation duration; GD = gaze duration; NFPF= number of fixations in the first pass; SFD = single fixation duration; SP = skipping probability; TT = total fixation duration.

After these analyses, we undertook Bayes factor analyses for linear mixed models to provide statistical support for the null effect of group as well as the interaction of group and type. Bayes factors for the full model (i.e., *BF_Full,_* the model containing the main effects of group, type and their interaction), the model with only main effects (i.e., *BF_Main_*) and the model without group effects and interaction (i.e., *BF_GroupBase_*) were calculated. We were able to evaluate the non‐significant interaction between group and type as well as the null effect of group by comparing the two pairs of models (*BF_Group_ = BF_Main_/BF_GroupBase_*; *BF_Interaction_* = BF*_Full_*/BF*_InterBase,_*) separately. The results of the Bayesian analyses supported the null hypothesis. A sensitivity analysis with different priors (i.e., 0.25, 0.5, and 1) also provided consistent results. The results of the Bayesian analyses are shown in Table 7.

**Table 7:** Bayesian factor values for each of eye movement measures for the target regions in experiment 2

Measures	FFD			GD			SFD			NFPF			TT			SP		
Prior	.25	.5	1	.25	.5	1	.25	.5	1	.25	.5	1	.25	.5	1	.25	.5	1
The whole four‐character region																		
Group	.23	.14	.04	.38	.24	.13	.27	.17	.10	.40	.25	.13	.45	.29	.15	.23	.12	.02
Interaction	.07	.02	.01	.09	.03	.01	.17	.06	.01	.08	.03	.08	.1	.03	.01	.08	.02	.01
First 2‐Character region																		
Group	.30	.17	.10	.30	.18	.10	.30	.17	.10	.31	.18	.10	.30	.18	.10	.28	.16	.06
Interaction	.07	.02	.01	.18	.05	.02	.18	.03	.03	.35	.12	.02	.10	.03	.01	.09	.03	.01
Last2‐Character region																		
Group	.28	.16	.08	.28	.16	.10	.28	.16	.10	.33	.18	.10	.85	.55	.30	.29	.16	.06
Interaction	.09	.03	.01	.14	.04	.01	.13	.04	.01	.17	.06	.01	.09	.08	.01	.07	.02	.01

Note

FFD = first fixation duration; GD = Gaze duration; SFD = single fixation duration; NFPF = number of fixations in the first pass; TT = total fixation duration; SFD = single fixation duration; SP = skip probability; SP = skipping probability.

#### Four character target region

There was a significant string type effect for FFD, GD and TT. Participants spent less time processing one‐word strings compared to ambiguous and two‐word strings (though this effect was not reliable on FFD between one‐word strings and two‐word strings). There was, however, no significant difference between the ambiguous and two‐word string types. We also found a similar effect for NFPF, with more fixations on two‐word and ambiguous strings compared to one‐word strings, though for this measure the difference between one‐word strings and two‐word strings was marginal. There was no reliable participant group effect or interaction between participant group and string type across all the measures. These results suggest that one‐word strings were much easier to process than ambiguous strings and two‐word strings, and that ambiguous strings were processed comparably to two‐word strings. These effects occurred regardless of the participant group’s off‐line segmentation preferences. That is, regardless of which participant group they were in, the majority of readers initially processed one‐word strings as a single word, the majority of readers initially processed two‐word strings as two separate words, and finally, the majority of readers processed the ambiguous words as two‐word strings. To reiterate, these on‐line processing preferences occurred regardless of participants’ own individual, off‐line segmentation biases. Thus, readers processed four‐character strings that are unambiguously a single word as a single lexical unit, and four‐character strings that are unambiguously 2 two‐character words as two separate words. Four‐character strings that are ambiguous between a single four‐character word and 2 two‐character words were more likely processed as two separate words, and again, this occurred regardless of a participant’s particular off‐line segmentation preference. These data suggest that readers might have a default strategy to segment ambiguous text as smaller rather than larger lexicalized units during natural Chinese reading, and this is not necessarily reflected in their off‐line segmentation preferences.

#### First two‐character region

In the analyses for this region, participants spent longer when reading two‐word strings compared with one‐word strings. They also made more fixations on ambiguous strings compared with one‐word strings. There was a marginal interaction between group and string type on NFPF such that one‐word segmenters made more fixations during first pass reading on the first two‐character region of ambiguous strings than that of one‐word strings (*t* = 3.45, *p *< .001). However, a Bayesian analysis supports a null interaction (see Table 7). These effects are very similar to those observed for the full four‐character region, and again, we interpret them to suggest that readers processed unambiguous stimuli as either 2 two‐character strings, or a single four‐character string as appropriate. More importantly, however, the results once again suggest that readers have a default preference and are, therefore, more likely to process ambiguous strings as 2 two‐character words. This holds regardless of the segmentation preferences they exhibit in an off‐line assessment.

#### Last two‐character region

In these analyses, we obtained significant effects for the FFD, GD, NFPF and TT measures such that reading times were shorter for one‐word strings compared to two‐word strings and ambiguous strings, and the fixation count in the first run of reading was also greater for one‐word strings compared with two‐word strings. Furthermore, readers had longer total reading times on two‐word strings than on ambiguous strings. The results mirror those obtained in the analyses for the previous regions and are consistent with the claim that readers processed the one‐word strings as single words, and the two‐word strings as two words, probably due to the difference in the syntactic category between the two types of strings. However, it remains the case that the results across the measures are, in the main, consistent with the suggestion that readers exhibit a preference to process ambiguous strings as two separate words regardless of the preferences they exhibit in relation to off‐line word segmentation.

#### Additional analysis

**Figure 4:**
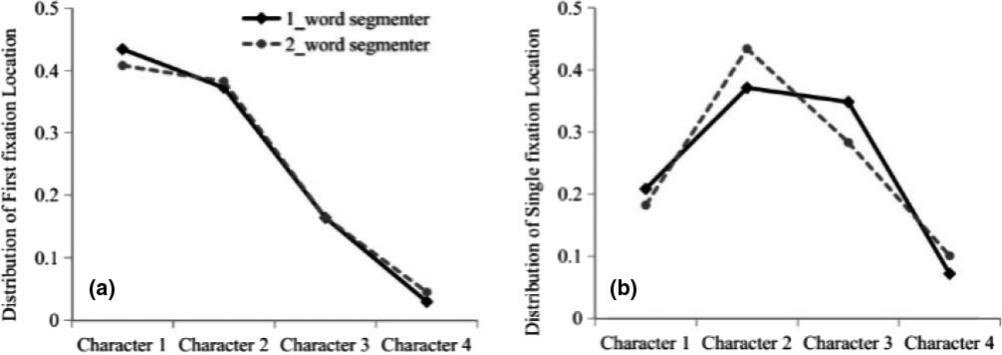
Proportions of first fixations and single fixations at four‐character positions for the one‐word and two‐word groups of segmenters.

An additional analysis was conducted to examine whether off‐line word segmentation preferences affected saccadic targeting during reading. If we consider single fixation landing position effects on characters two and three of the four‐character target string, then we should observe an interaction between participant group and character position, such that readers who are inclined to process the target string as 2 two‐character strings should have increased proportions of single fixations on the second character of the target relative to readers processing the string as a single four‐character word. In contrast, for readers inclined to process the string as a single four‐character word, there should be an increased proportion of single fixations on the third character of the target string compared with the participants processing the string as 2 two‐character words. We therefore analysed the landing position data on the four characters comprising the entire target region for the two‐word segmenters and the one‐word segmenters collapsing across the other experimental conditions (see Figure 4). Note, because very few single fixations landed on this portion of the four‐character target region, only 27.7% of the total number of trials were included in these analyses.

The interaction between segmenter group and character position was significant for characters 2 and 3 (*b *= .57, *SE *= .26, *z *= −2.17, *p *< .05). A planned contrast showed that the proportion of single fixations on the second character of the target string for the two‐word segmenters (*M *= 0.43, *SD *= 0.50) was the same as that of the one‐word segmenters (*M *= 0.37, *SD *= .48) (*b *= −0.27, *SE *= .18, *z *= 1.47, *p *> .05). In contrast, the proportion of single fixations on the third character of the target for the two‐word segmenters (*M *= 0.28, *SD *= 0.45) was marginally lower than the proportion for the one‐word segmenters (*M *= 0.35, *SD *= 0.48) (*b *= −.39, *SE *= .20, *z *= −1.91, *p *= .06). The pattern of single fixation landing positions was exactly as we predicted, Thus, we conclude that the word segmentation commitments that readers made as they processed the target string impacted not only reading times, but also saccadic targeting decisions.

## GENERAL DISCUSSION

In the present study, we investigated the correspondences between off‐line word segmentation and on‐line segmentation processing during Chinese reading. In Experiment 1, we selected a set of critical four‐character strings that were segmented by half of a group of participants as a single four‐character word, and by the remaining half of the group as 2 two‐character words. Then, a new group of participants were asked to read sentences which contained critical four‐character strings whilst their eye movements were recorded, and afterwards, they were required to segment the same sentences into words in an off‐line word segmentation task. For each item, participants were split into one‐word segmenters (who segmented four‐character strings as a single word) and two‐word segmenters (who segmented four‐character string as 2 two‐character words). Thus, we split participants into two groups (one‐word segmenters and two‐word segmenters) according to their off‐line segmentation bias. The data analysis showed no reliable group effect on all the eye movement measures.

In order to avoid the heterogeneity of participants and stimuli in Experiment 1, we undertook Experiment 2 to provide a stronger test. Specifically, in an off‐line segmentation study, we identified three sets of target character string stimuli. For the first set, almost all participants consistently segmented the string as a single four‐character word, and for the second set, almost all the participants consistently segmented the string as 2 two‐character words. Thus, for these stimuli, there was no variability across participants in relation to their segmentation preferences for each particular category of string. In addition, we identified a third set of stimuli, ambiguous stimuli, for which half of the participants segmented the strings as a single word, and half as 2 two‐character words. Upon selection of the items in each of our three categories of string, they were then each embedded in neutral sentence frames in readiness for our eye movement experiment to investigate segmentation preferences in reading. We pre‐screened a new set of participants to identify two groups; a group who exhibited a strong preference to segment four‐character strings as a single word, and a group who were inclined to segment the strings as 2 two‐character words. We then undertook our eye movement experiment in which we tested our two groups of participants using the three sets of stimuli. Unlike Experiment 1, Experiment 2 produced very robust effects such that we observed clear evidence for strong word segmentation effects during reading. We found that reading times were shorter for stimuli categorized as a single four‐character word, and longer for stimuli categorized as 2 two‐character words. We interpreted this basic result to indicate that when participants processed the string as two separate words, then they performed lexical identification twice, whereas when they processed the string as a single word, they engaged in this process only once. Thus, reading times were inflated for the 2 two‐character stimuli relative to the strings that were categorized as a single four‐character word. Another important and interesting aspect of our data was that the reading time data for the ambiguous target words very largely patterned in line with the data for the 2 two‐character words. Thus, it appears that in order to observe eye movement behaviour that reflects participants treating a four‐character string as a single word, then that string must be very widely perceived as a single word. That is to say, such effects will only occur when all the participants share the view that the string is a single word. In turn, this suggests that when character strings are ambiguous in that there are not strong preferences for one particular segmentation relative to another across a population of participants, then it is very likely that they have not (at least as yet) been lexicalized and represented in the mental lexicon as a single unit sufficiently widely across a participant population to observe systematic effects.

These findings are also in line with theoretical accounts of compound words such as the dual‐route (Pollatsek, Hyönä, & Bertram, 2000), multiple‐route (Kuperman, Schreuder, Bertram, & Baayen, 2009) and CAOSS (Composition as Abstract Operation in Semantic Space) ( Günther & Marelli, 2019, Günther, Marelli, & Bölte, 2020; Marelli, Gagne, & Spalding, 2017) models. The first two models posit that compound words are processed via both a compositional route, in which each constituent is identified separately and then combined to form the compound, and a direct lookup route that accesses a unified lexical entry for the whole compound. The recently proposed CAOSS model posits that compound processing involves activating the meaning representation of the constituents and the whole compound as separate units, but also involves a compositional process whereby the meaning of constituents may be combined to form a new representation. In other words, both the semantic relatedness between constituent meanings and the whole compound meaning, as well as the compositionality of constituent meanings (that is, the degree to which the compound meaning is predicted given its constituents), all play an important role in the compound processing. Presumably, both the compositional route and the direct lookup route are simultaneously active, at the lexical and semantic level. In the current study, it seems that the four‐character strings were processed via the direct lookup route, whilst the constituents of the 2 two‐character word strings and ambiguous combinations were each identified separately. Thus, the time spent processing 2 two‐character word strings and ambiguous strings was longer than the time spent processing a single four‐character word string that was very likely represented lexically. We note that further research is required to investigate whether the relatedness between meanings of two constituents and meanings of the combinations, or the compositionality of the constituent meanings in different types of strings affects how a multi‐constituent strings are processed during on‐line reading. To us, this represents an interesting and potentially informative future line of investigation.

Apart from the analysis of the whole four‐character region, we also analysed the data on the first two and last two characters and obtained very similar and consistent results compared with the whole region. The first two characters and the last two characters of the 1‐word strings were processed more quickly than the counterpart regions of two‐word and ambiguous strings. These results are consistent with the predictions of the model of Chinese word recognition and segmentation in reading proposed by Li et al. (2009). In this model, there are three levels of processing (a visual perception level, a character recognition level and a word segmentation and recognition level) that occur when a Chinese word is recognized. Also, the lower level processes by which a character is identified can be affected by word knowledge from the word level. For example, if a character is a sub‐unit of a word that itself is activated to a significant degree, then that sub‐unit will receive a boost to its activation from the word recognition level. In the current study, for the one‐word target strings, word segmentation and identification occur only once, whereas for the two‐word strings, it occurs twice (once for the first two‐character word, and then again for the second two‐character word). It is for this reason that reading times on target strings that comprised of a single word were shorter than those for two‐word and ambiguous strings. It is important to note, therefore, that implicit within this explanation is the assumption that when readers encounter a character string that is ambiguous between a single four‐character word and 2 two‐character words, they have a predisposition and are more likely to process the string as two separate words rather than a single word. To be clear, it appears that for ambiguous multi‐constituent strings that are likely not firmly established as representations in the lexicon, that is, they are not firmly lexicalized, then readers adopt a finer rather than a coarser grained level of analysis as they engage in identification and they do this regardless of their off‐line processing preferences.

The results from the two experiments together are informative at a number of levels. First, let us consider a methodological point. Our results demonstrate the importance of thinking very carefully about the design of an experiment if we are to capture differences in processing preferences across different participants and different stimuli. Clearly, the conclusions we may have reached on the basis of the results of the first experiment would have been very different to those we can form on the basis of the second. When considering variability in a dependent measure in relation to simultaneous variability in both participants and stimuli, it is critical to consider the consistency of effects in relation to both sources of variability. Second, pronounced effects in off‐line word segmentation studies do not necessarily predict robust and corresponding effects reflecting segmentation preferences during the computation of word boundaries during natural Chinese reading. From both experiments, we can see that the degree to which there is homogeneity of response across the entire participant set is a fundamental determinant of the extent to which readers exhibit a particular segmentation preference. Further, the results of Experiment 2 indicate that when almost all participants exhibit a particular off‐line segmentation preference for the same particular stimulus, then it is likely that eye movement data in reading experiments will also reflect that preference. However, when a roughly comparable proportion of participants each favour an alternative segmentation, based on the present findings, it is very likely that readers will homogeneously initially process an ambiguous string as individually separate, smaller, constituent units rather than a single larger multi‐constituent unit. To reiterate, for any strings that are not absolutely recognized as single multi‐constituent units that themselves are lexicalized, it appears that readers adopt a finer grained level of word segmentation analysis in relation to the linguistic units over which visual and linguistic processing is operationalized, and this finer grained segmentation adopted in preference to a more global level of analysis. This is an important point. It appears that a necessary condition for us to observe eye movement behaviour reflecting a more global, multi‐constituent unit, level of segmentation is a significant level of consensus across participants regarding the existence of that unit as a single lexical entity. It seems to be the case that if participants have any sense that a character string even *may be* comprised of two or more constituents, then they opt to process it as such, making fixations that reflect repeated lexical identification of individual constituent parts, rather than identifying the whole string as a single lexical unit in one operation. It is very tempting to conclude that the lexicalization of multi‐constituent units, that is, units that are comprised of multiple elements but represented as a single lexical representation (c.f., Cutter et al., 2014; see also Zang, 2019), is a process that occurs only after a very significant degree of exposure. It also appears that multi‐constituent lexical units cannot be said to exist unless there is a substantial level of quite broad (presumably implicit) agreement across the population in respect of their status.

Last but not least, our theorizing regarding multi‐constituent units, lexicalization, and the operationalization of visual and linguistic processing across eye movements during natural reading raises another important question that, at present, we can only speculate upon. Namely, what are the factors that determine whether a multi‐constituent unit in Chinese will be lexicalized and recognized via the identification of a single lexical representation, rather than via multiple individual lexical representations? At present, from our perspective, it is difficult to answer this question and further research is clearly required to elucidate the situation. A likely possibility, however, is that certain categories of multi‐word strings are very likely candidates to have multi‐constituent unit status. For example, intrinsic characteristics of strings such as having high co‐occurrence frequencies (Ellis, 2002; Wray, 2002), strings that have high predictability relationships such that later constituents are predictable based on earlier constituents (Cutter et al., 2014; Siyanova, 2010; the present results), mutual dependence, entropy or pointwise mutual information (PMI) (Hendrix & Sun, 2020) such that the meaning of the whole unit critically depends on shared aspects of meaning, etc., might all have a bearing on the likelihood that multi‐constituent units will be lexicalized and processed as single elements during reading. To reiterate, however, we fully acknowledge that further work is required before we can offer solid suggestions as to the factors that determine the likelihood that a multi‐constituent unit in Chinese might be processed as a single lexical item during natural reading.

In summary, the results of our experiments clearly showed that the relationship between off‐line word segmentation processes and on‐line word segmentation processes that occur during reading is complex and often not immediately transparent. Further, our results suggest that for multi‐character stimuli that are not widely recognized across a participant population as being single words, readers initially adopt a fine grained level of processing, segmenting them initially into their constituent parts. It appears that when readers do this, it impacts fixation times on words consistent with the suggestion that readers engage in successive episodes of lexical identification for each of those constituent parts. We also suggest that such processing may impact saccadic targeting decisions. In contrast, when multi‐character strings are widely recognized as being a single word, they are very likely lexicalized and represented as a single lexical unit. Under such circumstances, processing appears to be operationalized across the entire multi‐constituent unit resulting in a single lexical identification episode and reduced processing time.

## Conflict of interest

All authors declare no conflict of interest.

## Author contributions

Liyuan He (Conceptualization; Data curation; Formal analysis; Writing – original draft) Ziming Song (Data curation; Formal analysis; Investigation) Min Chang (Data curation; Formal analysis; Investigation) Chuanli Zang (Conceptualization; Supervision; Visualization; Writing – review & editing) Guoli Yan (Conceptualization; Funding acquisition; Project administration; Supervision) Simon P. Liversedge (Conceptualization; Supervision; Writing – review & editing).

## Data Availability

The data files and analysis scripts used in this study are available at: https://osf.io/dsf32/

## Footnotes

^1^
We conducted a further set of LMM analyses in which stroke complexity, co‐occurrence of two constituent words, and transitional probability of the four‐character string were included as covariates in the four‐character region analyses, and stroke complexity, frequency, and pointwise mutual information of the two‐character strings were included as covariates in the first and last two‐character region analyses. The basic pattern of results was very similar to those we report here, indicating that the effects we report here exist independent of influences from these extraneous variables.

^2^
As in Experiment 1, we conducted a further set of LMM analyses in which stroke complexity, co‐occurrence of two constituent regions, and transitional probability of the four‐character string were included as covariates in the four‐character region analyses, and stroke complexity, frequency, and pointwise mutual information of the two‐character string were included as covariates in the first and last two‐character region analyses. Again, the basic pattern of results was very similar to those we report here, indicating our effects exist independent of these extraneous influences.
